# Where Do AQP4 Antibodies Fit in the Pathogenesis of NMO?

**DOI:** 10.1155/2012/862169

**Published:** 2012-03-12

**Authors:** Makoto Kinoshita, Yuji Nakatsuji

**Affiliations:** Department of Neurology, Osaka University Graduate School of Medicine, D4, 2-2 Yamadaoka, Suita, Osaka 565-0871, Japan

## Abstract

Recent advances in the field of neuromyelitis optica (NMO) research provided convincing evidence that anti-AQP4 antibody (AQP4-Ab) not only serves as a highly specific disease marker, but also plays an essential role in the pathogenesis of the disease. Although it is now widely recognized that AQP4-Ab induces astrocytic necrosis in a complement-dependent manner, additional triggers are also suspected as a prerequisite for the development of the disease. Unraveling these unresolved aspects of the disease will provide substantial insight into still controversial issues in the pathogenesis of NMO.

## 1. Introduction

During the past several years there has been a huge progress in our understanding of the pathogenesis of neuromyelitis optica (NMO). The discovery of a disease-specific autoantibody, anti-aquaporin-4 antibody (AQP4-Ab), in the sera of patients with NMO has attracted enormous attention of researchers within the field [1, 2]. Among a large number of reports related to the pathogenesis of NMO, animal studies have provided substantial insight into the pathogenic mechanism of AQP4-Ab [3–6]. In this review article, we discuss the current view of the pathogenic mechanism of NMO based upon the studies of AQP4-Ab, and further point out the unresolved issues related to the pathogenesis of NMO.

## 2. AQP4-Ab is Not Only a Disease Marker but a Pathogenic Autoantibody

Since the identification of a highly disease-specific autoantibody, AQP4-Ab, in the sera of patients with NMO, several clinical observations suggested the pathogenicity of AQP4-Ab [1, 2]. It has been widely appreciated that the therapeutic intervention by plasmapheresis is a beneficial treatment of patients with NMO [7, 8]. The disease activity is also reported to correlate with the titer of AQP-Ab in the serum or the CNS [9–11]. More importantly, the active lesions of NMO are characterized by the loss of AQP4 and glial fibrillary acidic protein (GFAP) immunoreactivities (IRs) [12, 13]. In addition to these clinical observations, the direct evidence of the pathogenicity of AQP4-Ab was recently provided by *in vitro* and *in vivo* studies. It is reported from several groups that AQP4-Ab-positive sera induce necrosis of astrocytes in a complement-dependent manner *in vitro* [14–16]. Another mechanism of Ab-dependent cellular cytotoxicity (ADCC) is also suggested in assays using human astrocytes [17]. Most importantly, we and others have shown that when rats were preimmunized with myelin basic protein (MBP) and experimental autoimmune encephalomyelitis (EAE) was induced, injection of immunoglobulins collected from patients with NMO can induce strikingly similar pathological features to NMO in the recipient rats [3–5]. The active lesions of these models were characterized by the extensive loss of AQP4 and GFAP-IRs especially around the blood vessels and meninges where AQP4 is predominantly expressed [3–5]. The specific deposition of activated complement and transferred immunoglobulins at the sites of astrocytic loss was reminiscent of the NMO patients' pathology [4, 5]. It is noteworthy that, at the borders of astrocytic loss in this animal model, more extensive loss of AQP4-IR compared to GFAP-IR was observed, supporting the specificity of AQP4 as a target in this disease model [5]. The specificity of AQP4-Ab was also confirmed by either absorbing AQP4-Ab with AQP4-expressing cells or establishing monoclonal antibodies [3, 4]. These observations together have provided convincing evidence that AQP4-Ab is pathogenic both *in vitro* and *in vivo* and plays a pivotal role in the pathogenesis of NMO.

## 3. Astrocytic Necrosis Is Induced by AQP4-Ab in a Complement-Dependent Manner

Apart from the remarkable loss of astrocytes in NMO, the active lesions are also characterized by the deposition of activated complement and immunoglobulins [18]. The majority of AQP4-Ab produced in the sera of patients with NMO belongs to IgG1 isotype [19], which are the most potent immunoglobulin subclass capable of activating complement system. These clinical observations highly suggest that complement system is another essential factor in the pathogenesis of NMO. The important role of complement system during the astrocytic death caused by AQP4-Ab was also supported by animal studies. The lesions of astrocytic loss in the recipient rats given immunoglobulins of patients with NMO were accompanied by remarkable deposition of activated complement or C5b-9 [4, 5, 20]. Moreover, a C1 complement inhibitor is also reported to prevent the pathogenic effect of AQP4-Ab *in vivo* [6]. Similar inhibitory effect was also observed with cobra venom factor (CVF) on astrocytic death in our animal model (unpublished data). CVF is a widely used reagent that transiently depletes the active components of complement *in vivo*. When recipient rats were pretreated with CVF, the loss of GFAP-positive cells in the spinal cords was much less observed. *In vitro* studies also showed that AQP4-Ab-positive sera are capable of inducing astrocytic death only in the presence of complement [14, 15]. Furthermore, the type of astrocytic death caused by AQP4-Ab was shown to be necrosis induced by immune complexes of C5b-9 [14]. When rat primary astrocytes were incubated with AQP4-Ab-positive sera, most of the dying astrocytes became positive both for Annexin V and PI, the pattern of staining suggesting the necrotic process in the target cells. Immunocytochemistry of these cells showed that there is a deposition of C5b-9 on dying astrocytes [14]. These observations may partially explain why the active lesions in NMO are characterized by highly destructive features of necrosis [18].

## 4. Do AQP4-Abs Become Pathogenic Once in the Brain?

Although the passive transfer models of NMO confirmed the pathogenicity of AQP4-Ab on astrocytes, it still remains unclear whether AQP4-Ab is a disease-modifying factor or a primary initiator of the disease [21]. Since AQP4-Ab does not penetrate the blood-brain barrier (BBB) under physiological condition [4], there should be at least the second trigger to break the integrity of the BBB and let AQP4-Ab get an access to their target antigen in the CNS. But is the disruption of the BBB enough for AQP4-Ab to exert its pathogenicity to full extent? Though there are some anatomical sites where the BBB is relatively loose and permeable [22], most of the patients who harbor AQP4-Ab in their sera do not show any clinical symptoms during remissions [23]. In addition, there is a report of a case that showed positivity of AQP4-Ab more than 10 years before the onset of the disease [24]. Unlike myasthenia gravis, the most well-characterized autoantibody-mediated disease, maternal transmission of the disease during pregnancy has not been reported in NMO [25]. *In vivo* studies also suggest that pathological changes are not reproducible even after transferring patients' immunoglobulins to juvenile naïve rats where the integrity of BBB is still fragile. Therefore, the access of AQP4-Ab beyond the BBB is presumably not sufficient to induce the astrocytic cytotoxicity [4]. This hypothesis might seem to contradict with the recent report that showed the pathogenic effect of AQP4-Ab after being directly injected into mouse brains with human complement [6]. This discrepancy, however, may be explained by the fact that astrocytes are endowed with various types of complement-regulatory proteins (CRPs), and these CRPs are known of being not capable of playing their protective role against activated complement of different species [26, 27]. Since the loss of astrocytes was only observed with human complement but not with mouse complement in the report, the interpretation of the results in this disease model should be carefully made [6]. Taken together, we speculate that the second trigger besides the leakage of BBB might be required for AQP4-Ab to become fully pathogenic.

## 5. Nonspecific Inflammation Potentiates the Full Pathogenicity of AQP4-Ab in the Absence of T Cells

If the leakage of BBB is not sufficient for AQP4-Ab to become fully pathogenic, then what other factors are necessary? We have recently shown that pretreatment of recipient rats with complete Freund's adjuvant (CFA) alone is sufficient for the AQP4-Ab transferred in the periphery to induce astrocytic cytotoxicity in the spinal cords [20]. CFA is a commonly used reagent to induce various models of autoimmune diseases [28]. CFA activates innate immune cells and leads to the release of various types of inflammatory cytokines [28]. This nonspecifically induced inflammation in the periphery not only disrupts the integrity of the BBB but also activates glial cells in the CNS [29]. In our CFA model, T cells are scarcely observed in the lesions [20]. Therefore, at the very first step of the development of NMO, nonspecific inflammation induced in the periphery is sufficient to potentiate the pathogenicity of AQP4-Ab and T cells are not necessarily required [20]. It remains to be elucidated whether the alteration of inflammatory status in the periphery or that in the CNS induced after the injection of CFA is important to break the tolerance. It may be that complement system gets excessively activated, rendering astrocytes vulnerable to the attack from immune complexes. Or alternatively, expression pattern of CRPs might be altered on astrocytes after the stimulation with CFA. Another possibility is that inflammatory cytokines produced in the CNS alter the expression pattern or level of AQP4 on astrocytes [30], providing more easily accessible target antigens to AQP4-Ab. Future studies should address this question by examining the profile of inflammatory cytokines and complement-related molecules, along with the change in the pattern of AQP4 expression within the CNS.

## 6. Are T Cells Also Responsible for the Development of the Disease?

Although we have learned through our animal model that nonspecific inflammation is sufficient to induce the pathogenic effect of AQP4-Ab, it is noteworthy that the lesions observed in CFA model were much less extensive compared to those of recipient rats preimmunized with MBP (EAE model) [5, 20]. Only with high titer of AQP4-Ab the loss of astrocytes was observed in CFA model while with antibodies of lower titer only the ballooning of astrocytes was observed [20]. Taken into account that the active lesions of NMO also contain substantial number of T cells, T cells will also affect the disease activity in NMO [18]. Recent reports showed that certain amino sequence of AQP4 is immunogenic to induce AQP4-specific T cells in several animal strains [31, 32]. More importantly, it is documented that patients with NMO harbor activated T cells specific for AQP4 in the periphery [33]. It is also reported that there is an increase in Th1 and Th17 subsets in the periphery of NMO spectrum disorders [34–36]. These observations suggest that pathogenic T cells against AQP4 do exist in the periphery of patients with NMO and might also accelerate the disease activity once they encounter the target antigen in the CNS. Whether these T cells provide an initial trigger before the entry of AQP4-Ab remains to be elucidated. Of note, it is also reported that patients with NMO harbor the T cells specific for other antigens comprising the CNS, such as MBP and proteolipid protein [21, 33]. Where these T cells fit in the picture of NMO pathogenesis is hard to conclude. These T cells are probably induced by well-recognized phenomena of antigen spreading after tissue destruction by AQP4-Abs. Although less likely, however, we cannot exclude the possibility that these T cells are initially activated at the very first stage of the disease and AQP4-targeted inflammation occurs afterwards. Notably, less than 30% of actively demyelinating lesions show AQP4 loss in NMO [21].

## 7. T Cells and the Location of the Inflammation in NMO

**Figure 1:**
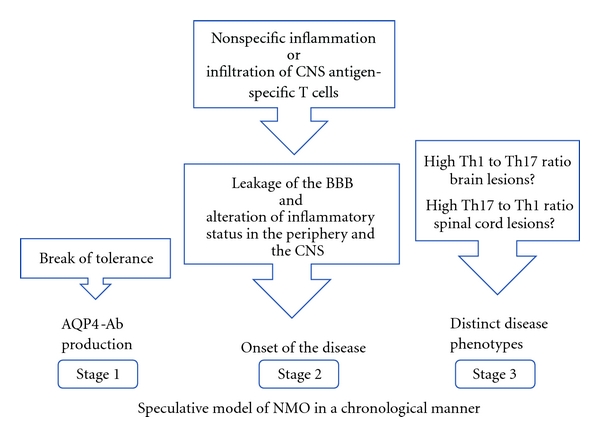
Speculative model of AQP4-Ab-positive NMO is shown in a chronological manner. At the very first stage of the disease course, break of tolerance against AQP4 leads to production of AQP4-Ab in the periphery without manifesting any symptoms. What causes the compromise of the tolerance is not yet elucidated. Molecular mimicry of AQP4 to certain microbes is one of the possibilities. Onset of the disease might be triggered by either nonspecific inflammation caused by infections or activation of CNS antigen-specific T cells. These stimuli will not only induce the leakage of the BBB, but also alter the inflammatory status both in the periphery and within the CNS. Once the disease develops, the location of the lesions may be determined by the types of T cells infiltrating within the CNS. The presence of AQP4-Ab will further modulate the disease phenotype and produce characteristic lesions of NMO spectrum disorders.

The prototypical presentation of NMO is characterized by preferential involvement of central gray matter in the spinal cord and brain stems, where AQP4 is known to be expressed at high level [22]. Recent clinical reports, however, provide numerous lines of evidence that nonspecific cerebral lesions are not as rare as used to be recognized [37]. The change in this clinical perception of the disease apparently counteracts with the notion that AQP4-Ab is the only factor blamed for inducing inflammation in the disease. To give rationale explanation for this discrepancy, we might need to pay more attention to the role of T cells in the pathogenesis of NMO. In addition to AQP4-Ab, T cells might play an important role in deciding the location of lesions in the CNS. Recently it is reported from several groups that Th1 cells and Th17 cells, the two major proinflammatory T cell subsets, induce distinct phenotypes of inflammation in EAE models [38, 39]. It is interesting that large number of children who show positive results of AQP4-Ab present with symptomatic brain involvement [40]. This might be explained by the hypothesis that the balance between Th1 and Th17 cells affects the clinical presentation of the disease. Taking into account the fact that patients with opticospinal involvement show increase in the production of IL-17 within the CNS, it might be that patients present inflammation in the spinal cords and not in the brains when Th17 cells become predominant [41]. The exact clinical phenotype induced by Th1 immunity is not yet clear (see Figure 1). This hypothesis regarding the balance of inflammatory T-cell subsets in defining the disease location might further be extended to explain why some proportion of patients with typical NMO presentation turns out to be negative for AQP4-Ab [37]. The passive transfer of immunoglobulins collected from these so-called seronegative NMO patients is reported not to induce astrocytic loss in an animal model [4]. This raises the question as to whether the so-called seronegative NMO is truly an autoantibody-mediated disease or not. Given that some patients with seronegative NMO respond to plasmapheresis, and others become seropositive during the disease course, heterogenic pathogenesis is postulated [42, 43].

## 8. Granulocyte Involvement in the Pathogenesis of NMO

In spite of the huge progress in the mechanism of AQP4-Ab-related pathology, much less is investigated as to how granulocytes recruited in the active lesions of NMO are related to the disease [18]. The granulocytes are generally considered to be recruited by components of activated complement, such as C5a and C3a [17]. Besides the involvement of complement in our disease model [20], we recently observed that even in the absence of T cells there is an increase in the expression of CINC-1, a functional homolog of human IL-8, in the spinal cords of recipient rats (unpublished data). Considering the fact that increase of IL-8 production is reported in the spinal cord of patients with opticospinal manifestation [41], IL-8 produced by astrocytes might be another essential factor attracting granulocytes in the lesions [44, 45]. To what extent these recruited granulocytes are involved in the pathogenesis of NMO still remains to be elucidated. *In vitro* studies did not confirm the pathogenic effect of activated granulocytes on astrocytes even after they showed degranulation [17]. More detailed studies *in vivo* should elucidate whether granulocytes are actively involved in the formation of pathology in NMO.

## 9. Conclusions

Recent progress of animal studies has provided substantial insight into our knowledge of NMO pathogenesis. Nonpathogenic recombinant monoclonal anti-AQP4 Ab, called “aquaporumab,” was recently shown to dramatically reduce NMO-like lesions in an *ex vivo* spinal cord slice model [46]. Although there is no doubt that AQP4-Ab plays a pivotal role in the development of the disease, AQP4-Ab alone cannot explain the whole spectrum of the disease manifestations. The absence of pathological changes in the peripheral organs expressing AQP4 despite the rapid access of AQP4-Ab supports this hypothesis [47]. The correlation of serum AQP4-Ab titer and the patients' disability or relapse rates was also challenged in recent reports [21, 48]. We suspect that there should be additional triggers to potentiate the pathogenicity of AQP4-Ab to its full extent. Among the additional triggers to be considered, inflammatory T-cell subsets should be paid extra attention. Unraveling the exact role of T cells along with that of recruited granulocytes will provide deeper understating of the disease mechanism of patients with NMO spectrum disorders.
